# Linzagolix therapy versus a placebo in patients with endometriosis-associated pain: a prospective, randomized, double-blind, Phase 3 study (EDELWEISS 3)

**DOI:** 10.1093/humrep/deae076

**Published:** 2024-04-22

**Authors:** Jacques Donnez, Christian Becker, Hugh Taylor, Francisco Carmona Herrera, Olivier Donnez, Andrew Horne, Maciej Paszkowski, Felice Petraglia, Stefan P Renner, Amisha Patel, Mitra Boolell, Elke Bestel, Marie-Madeleine Dolmans

**Affiliations:** Department of Gynecology, Université Catholique de Louvain, Brussels, Belgium; Department of Gynecology, Société de Recherche pour l’Infertilité (SRI), Brussels, Belgium; Nuffield Department of Women’s & Reproductive Health, Endometriosis CaRe Centre, University of Oxford, Oxford, UK; Department of Obstetrics, Gynecology and Reproductive sciences, Yale School of Medicine, New Haven, CT, USA; Gynaecology Department, Clinic Institute of Gynaecology, Obstetrics and Neonatology (ICGON), Hospital Clinic of Barcelona, Universitat de Barcelona, Barcelona, Spain; Département de Gynécologie, Centre de l’Endométriose Complexe, Chirurgie endoscopique pelvienne, Polyclinique Urbain V (ELSAN Group), Avignon, France; Department of Gynecology, EXPPECT and MRC Centre for Reproductive Health, University of Edinburgh, Edinburgh, UK; Third Chair and Department of Gynecology, Medical University of Lublin, Lublin, Poland; Obstetrics and Gynecology Unit, Department of Clinical Experimental and Biomedical Sciences, University of Florence, Florence, Italy; Department of Gynecology and Obstetrics, Hospital Böblingen, Klinikverbund-Suedwest, Sindelfingen 71065, Germany; Department of Medical Affairs, Theramex UK Ltd, London, UK; Department of Medical Affairs, Theramex UK Ltd, London, UK; Department of Medical Affairs, Theramex UK Ltd, London, UK; Gynecology Research Laboratory, Institut de Recherche Expérimentale et Clinique, Department of Gynecology, Université Catholique de Louvain, Brussels, Belgium; Gynecology Department, Cliniques Universitaires St-Luc, Brussels, Belgium

**Keywords:** endometriosis, oral GnRH antagonist, linzagolix, non-menstrual pelvic pain, dysmenorrhea, endometriosis-associated pain

## Abstract

**STUDY QUESTION:**

Does linzagolix administered orally once daily for up to 3 months at a dose of 75 mg alone or 200 mg in combination with add-back therapy (ABT) (1.0 mg estradiol; 0.5 mg norethindrone acetate, also known as norethisterone acetate [NETA]) demonstrate better efficacy than placebo in the management of endometriosis-related dysmenorrhea and non-menstrual pelvic pain?

**SUMMARY ANSWER:**

Combining 200 mg linzagolix with ABT was found to significantly reduce dysmenorrhea and non-menstrual pelvic pain at 3 months of therapy, while a daily dose of 75 mg linzagolix yielded a significant decrease only in dysmenorrhea at 3 months.

**WHAT IS KNOWN ALREADY?:**

A previously published Phase 2, dose-finding study reported that at a dose of 200 mg daily, linzagolix promotes full suppression of estradiol secretion to serum levels below 20 pg/ml and noted that the addition of ABT may be needed to manage hypoestrogenic side effects. At lower doses (75 mg and 100 mg/day), linzagolix maintains estradiol values within the target range of 20–60 pg/ml, which could be ideal to alleviate symptoms linked to endometriosis.

**STUDY DESIGN, SIZE, DURATION:**

EDELWEISS 3 was a multicenter, prospective, randomized, placebo-controlled, double-blind, double-dummy Phase 3 study to evaluate the safety and efficacy of linzagolix for the treatment of moderate-to-severe endometriosis-associated pain. Treatment was administered orally once daily for up to 6 months.

**PARTICIPANTS/MATERIALS, SETTING, METHODS:**

In the EDELWEISS 3 trial, 486 subjects with moderate-to-severe endometriosis-associated pain were randomized at a 1:1:1 ratio to one of the three study groups: placebo, 75 mg linzagolix alone or 200 mg linzagolix in association with ABT. Pain was measured daily on a verbal rating scale and recorded in an electronic diary.

**MAIN RESULTS AND THE ROLE OF CHANCE:**

At 3 months, the daily 200 mg linzagolix dose with ABT met the primary efficacy objective, showing clinically meaningful and statistically significant reductions in dysmenorrhea and non-menstrual pelvic pain, with stable or decreased use of analgesics. The proportion of responders for dysmenorrhea in the 200 mg linzagolix with ABT group was 72.9% compared with 23.5% in the placebo group (*P* < 0.001), while the rates of responders for non-menstrual pelvic pain were 47.3% and 30.9% (*P* = 0.007), respectively. The 75 mg linzagolix daily dose demonstrated a clinically meaningful and statistically significant reduction in dysmenorrhea versus placebo at 3 months. The proportion of responders for dysmenorrhea in the 75 mg linzagolix group was 44.0% compared with 23.5% in the placebo group (*P* < 0.001). Although the 75 mg dose showed a trend toward reduction in non-menstrual pelvic pain at 3 months relative to the placebo, it was not statistically significant (*P* = 0.279). Significant improvements in dyschezia and overall pelvic pain were observed in both linzagolix groups when compared to placebo. Small improvements in dyspareunia scores were observed in both linzagolix groups but they were not significant. In both groups, hypoestrogenic effects were mild, with low rates of hot flushes and bone density loss of <1%. A daily dose of 200 mg linzagolix with ABT or 75 mg linzagolix alone was found to significantly reduce dysmenorrhea and non-menstrual pelvic pain also at 6 months of therapy.

**LIMITATIONS, REASONS FOR CAUTION:**

Efficacy was compared between linzagolix groups and placebo; however, it would be useful to have results from comparative studies with estro-progestogens or progestogens. It will be important to ascertain whether gonadotropin-releasing hormone antagonists have significant benefits over traditional first-line medications.

**WIDER IMPLICATIONS OF THE FINDINGS:**

Linzagolix administered orally once daily at a dose of 200 mg in combination with add-back therapy (ABT) demonstrated better efficacy and safety than placebo in the management of moderate-to-severe endometriosis-associated pain. The quality of life was improved and the risks of bone loss and vasomotor symptoms were minimized due to the ABT. The 75 mg dose alone could be suitable for chronic treatment of endometriosis-associated pain without the need for concomitant hormonal ABT, but further research is needed to confirm this. If confirmed, it would offer a viable option for women who do not want to wish to have ABT or for whom it is contraindicated.

**STUDY FUNDING/COMPETING INTEREST(S):**

Funding for the EDELWEISS 3 study was provided by ObsEva (Geneva, Switzerland). Analysis of data and manuscript writing were partially supported by ObsEva (Geneva, Switzerland), Theramex (London, UK) and Kissei (Japan) and grant 5/4/150/5 was awarded to M.-M.D. by FNRS. J.D. was a member of the scientific advisory board of ObsEva until August 2022, a member of the scientific advisory board of PregLem, and received personal fees from Gedeon Richter, ObsEva and Theramex. J.D. received consulting fees, speakers’ fees, and travel support from Gedeon Richter, Obseva and Theramex, which was paid to their institution. C.B. has received fees from Theramex, Gedeon Richter, and Myovant, and travel support from Gedeon Richter—all funds went to the University of Oxford. He was a member of the data monitoring board supervising the current study, and served at an advisory board for endometriosis studies of Myovant. H.T. has received grants from Abbvie and was past president of ASRM. F.C.H. has received fees from Gedeon Richter and Theramex. O.D. received fees for lectures from Gedeon Richter and ObsEva and research grants for clinical studies from Preglem and ObsEva independent from the current study. A.H. has received grants from NIHR, UKRI, CSO, Wellbeing of Women, and Roche Diagnostics; he has received fees from Theramex. A.H.’s institution has received honoraria for consultancy from Roche Diagnostics, Gesynta, and Joii. M.P. has nothing to declare. F.P. has received fees from Theramex. S.P.R. has been a member of the scientific advisory board of Gedeon Richter and received fees from Gedeon Richter. A.P. and M.B. are employees of Theramex. E.B. was an employee of ObsEva, sponsor chair of the data monitoring board supervising the current study, and has been working as a consultant for Theramex since December 2022; she owns stock options in ObsEva. M.-M.D. has received fees and travel support from Gedeon Richter and Theramex.

**TRIAL REGISTRATION NUMBER:**

NCT 03992846.

**TRIAL REGISTRATION DATE:**

20 June 2019.

**DATE OF FIRST PATIENT’S ENROLLMENT:**

13 June 2019.

## Introduction

Endometriosis is a chronic inflammatory disorder affecting almost 10% of women over the course of their reproductive years, causing various pain symptoms like dysmenorrhea, chronic non-menstrual pelvic pain, dyspareunia, and dyschezia and, in some instances, dysuria and painful urination (Giudice and Kao, 2004; Parazzini *et al*., 2020; Zondervan *et al.*, 2020; Donnez and Dolmans, 2021a,b; Bonavina and Taylor, 2022; Donnez and Cacciottola, 2022; Horne and Missmer, 2022). Endometriosis is also a leading cause of infertility and is often identified during fertility investigations. Endometriosis-associated pain symptoms can seriously impact the quality of life in women with the disease, often resulting in absenteeism and loss of productivity at work (Pluchino *et al.*, 2016; Barbara *et al.*, 2021). Indeed, it constitutes a substantial economic burden and considerable financial outlay for society and health insurance systems (Gao *et al.*, 2006; Simoens *et al.*, 2012; Soliman *et al.*, 2016, 2017). The World Endometriosis Research Foundation estimated the combined annual costs of endometriosis to be around $80 billion in the USA and $60 billion in the UK, France, Germany, and Italy in 2012 taking into account exchange rates at the time (Soliman *et al.*, 2018).

Progression of endometriotic lesions is estrogen-dependent, and guidelines issued by ESHRE, (2022), ASRM (2014), and NICE guideline [NG73] (2017) recommend long-term treatment by inhibiting ovulation or reducing estradiol (E2) production (Dunselman *et al.*, 2014; Practice Committee of the American Society for Reproductive Medicine, 2014; NICE guideline [NG 73], 2017; Becker *et al.*, 2022; Vannuccini *et al.*, 2022). While existing therapies for endometriosis are either medical or surgical (Donnez and Dolmans, 2021a,b), identification of the most appropriate medical therapy is challenging, as the existing treatments have certain limitations (Zondervan *et al.*, 2020; Cacciottola *et al.*, 2021; Taylor *et al.*, 2021). Medical therapy should ideally aim to relieve pain or reduce the lesion size (Cacciottola *et al.*, 2021).

First-line treatments for endometriosis, such as combined oral contraceptives (COCs) and progestogens, work by stopping ovulation and lessening menstrual bleeding. However, they prove effective for only about two-thirds of women dealing with pain related to endometriosis (Vercellini *et al.*, 2016, 2018a,b). Despite many experts advocating for progestogens as the primary choice of treatment (Casper, 2017), there’s a notable failure rate of ∼33%, largely attributed to what is known as progesterone resistance (Bulun *et al.*, 2019, 2023; Yilmaz and Bulun, 2019; Taylor *et al.*., 2021; Donnez and Dolmans, 2021a). The notion of progesterone resistance was first proposed in 1997 (Nisolle and Donnez, 1997), and since then, numerous papers have produced evidence to substantiate this hypothesis (for review, see Donnez and Dolmans, 2021a). According to Flores *et al.* (2018), Yilmaz and Bulun (2019), Bulun *et al.* (2019, 2023), Reis *et al.* (2020), and Donnez (2021), the inability of endometriotic stromal cells to produce progesterone-induced paracrine factors could stem from a deficiency in progesterone receptor B (PR-B).

Second-line treatments (injectable depot formulations of gonadotropin-releasing hormone [GnRH] agonists) are generally only offered if COC or progestogens fail, as they are associated with menopausal symptoms (Barbara *et al.*, 2021; Becker *et al.*, 2022) such as bone mineral density (BMD) loss and hot flushes. While these drugs may alleviate endometriosis symptoms, they also come with significant drawbacks (Vercellini *et al.*, 2018b; Barbara *et al.*, 2021; Vannuccini *et al.*, 2022; Donnez and Dolmans, 2021a,b; Cacciottola *et al.*, 2021; Donnez *et al.*, 2022). They include the need for injection; the initial agonist or flare-up effect that can exacerbate the disease; full suppression of E2 to postmenopausal levels generally requiring add-back therapy (ABT) to counterbalance the adverse effects of total estrogen suppression, especially BMD loss; and finally the unpredictable length of time required to normalize ovarian function. Indeed, after the end of therapy, it can take months for ovarian function to return to normal.

Surgical excision or ablation of endometriotic lesions can also be effective for relief of pain (Donnez *et al.*, 2002). Nevertheless, lesions may recur, with symptoms worsening over time, requiring repeated courses of medical pain management or surgeries (Bozdag, 2015; Capezzuoli *et al.*, 2021).

Certainly, there is a significant unmet requirement for novel medical treatments for endometriosis. According to the estrogen threshold hypothesis proposed by Barbieri (1992), complete suppression of estrogen may not be necessary. Regulating estrogen levels to diminish menopausal symptoms, while preserving effectiveness in terms of reduction of endometriosis-related pain, represents a potentially appealing treatment alternative (Donnez *et al.*, 2017).

Oral GnRH antagonists have emerged as a potential alternative to allow dose-dependent regulation of E2 levels (Donnez *et al.*, 2017). In two similar double-blind, randomized, Phase 3 trials, Taylor *et al.* (2017) has demonstrated that elagolix, an oral non-peptide GnRH antagonist, administered at doses of 150 mg once daily and 200 mg twice daily, is effective at treating endometriosis-associated pain. Elagolix has a short half-life of ∼4–6 h, so twice-daily administration of the higher dose is required to achieve full suppression of endogenous estradiol production (Ng *et al.*, 2017). In two very recent replication Phase 3, randomized, double-blind studies, Giudice *et al.* (2022) reported that relugolix combination therapy (40 mg relugolix plus ABT: 1 mg E2 plus 0.5 mg NETA) significantly improved endometriosis-associated pain.

Linzagolix is an oral non-peptide GnRH antagonist with low pharmacokinetic/pharmacodynamic (PK/PD) variability (Pohl *et al.*, 2020, 2022). Like elagolix and relugolix, it works by binding to and blocking the GnRH receptor in the pituitary gland, resulting in a dose-dependent decline in the production of LH and FSH (Donnez *et al.*, 2017, 2020, 2022, 2023). At suitable doses, linzagolix has been shown to maintain E2 values within the desired range of 20–60 pg/ml, which might be optimal for alleviating symptoms associated with endometriosis, while simultaneously reducing BMD loss and other undesirable effects linked to complete E2 suppression (Donnez *et al.*, 2017, 2020). At increased doses, linzagolix decreases E2 levels to below 20 pg/ml, which is regarded as full suppression (Donnez *et al.*, 2017, 2020). Linzagolix was recently authorized by the European Commission for the treatment of myoma-related heavy menstrual bleeding and, in Europe, it has become the sole GnRH antagonist employing a non-hormonal strategy without ABT to satisfy the requirements of women who cannot or choose not to take hormones. Linzagolix is the only oral GnRH antagonist approved without ABT in Europe. At a dose of 100 mg, it was approved by EMA for management of uterine fibroids. Relugolix CT (with ABT) is the only alternate GnRH antagonist in commercial use in Europe. In the USA, 100 mg elagolix once a day and 200 mg twice daily (limited to 6 months) is approved for the treatment of endometriosis.

In 2019, the Phase 2b EDELWEISS 1 clinical trial was completed in patients with endometriosis, having enlisted 328 subjects with moderate-to-severe endometriosis-related pain who were allocated to one of six treatment groups, including oral placebo and fixed linzagolix doses of either 50, 75, 100, and 200 mg per day (Donnez *et al.*, 2020). This study demonstrated that linzagolix significantly reduced endometriosis-associated pain and enhanced quality of life at doses of 75 mg and above; however, the treatment led to a dose-dependent decrease in BMD (Donnez *et al.*, 2020). From this Phase 2b study, two doses (75 mg alone and 200 mg plus ABT) were selected for the Phase 3 study described here. We report the efficacy and safety of 24 weeks of once daily oral linzagolix in women with endometriosis-associated pain.

## Materials and methods

### Trial design and overview

**Figure 1.**
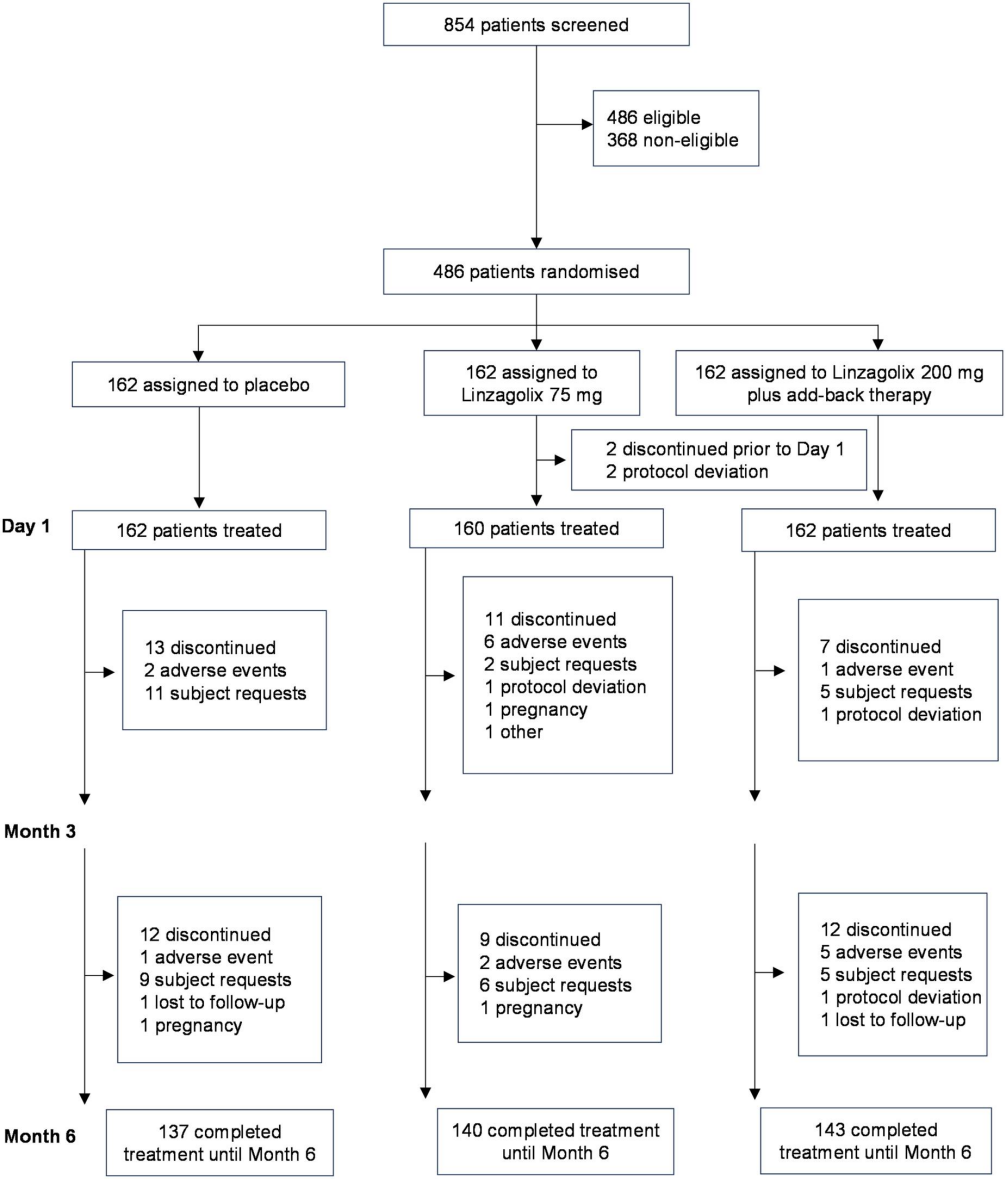
Subject disposition between screening, randomization, and study completion to Month 6.

EDELWEISS Phase 3 was a multinational, multicenter, prospective, randomized, placebo-controlled, double-blind, double-dummy trial conducted in 91 clinical centers in the USA (n = 26) and Europe (n = 65). The trial flow diagram is shown in Fig. 1. The study comprised a 3-month screening period, a 6-month treatment period, and a 6-month drug-free follow-up period. After eligibility was confirmed during the screening period based on at least two full menstrual cycles, subjects were randomized at a 1:1:1 ratio to one of the three study groups: 75 mg linzagolix alone, 200 mg linzagolix in combination with ABT (1.0 mg E2/0.5 mg NETA), or a placebo. Treatment was administered orally once a day for up to 6 months.

The trial was granted approval from the responsible ethics committee or institutional review board from each participating center and was carried out in accordance with the International Conference on Harmonization guidelines, applicable regulations, and ethical principles derived from the Declaration of Helsinki. The trial was recorded on ClinicalTrials.gov with the identifier NCT 03992846. All prospective study participants provided informed written consent before any screening activities. The sponsor, ObsEva S.A., was responsible for designing the trial.

To maintain consistency in such large multinational, geographically diverse trials, the sponsor involved a global Contract Research Organization (Lapcorp drug development INC) to identify investigational sites, set up the countries and locations, oversee data collection, and conduct site monitoring, ensuring that the same procedures and standards were used for all countries and sites. The data were analyzed by a different Contract Research Organization (Cytel INC), which similarly used identical rules for all countries/sites involved. Last but not least, the study sponsor oversaw the Contract Research Organizations and ensured consistency in decisions at sponsor level. The investigators, contract research organization and sponsor conducted the trial and gathered the data jointly.

The first draft of the manuscript was written by two medical doctors (J.D. and M.M.D.) and all the authors provided feedback on all versions of the manuscript. They all can attest to the completeness and accuracy of the data and analyses, and compliance with the trial protocol.

### Subjects

Eligible subjects were women of reproductive age with surgically confirmed pelvic endometriosis in the previous 10 years. They were enrolled at study centers in the USA and Europe. Prior to screening, the subjects needed to exhibit moderate-to-severe endometriosis-associated pain, defined as a score of at least two for dysmenorrhea and at least two for non-menstrual pelvic pain over the previous month according to the modified Biberoglu and Behrman (Mb&B) scale (Biberoglu and Behrman, 1981). During the screening period, over the course of two full menstrual cycles, subjects had to show a mean overall pelvic pain score of at least four (0–10 numeric rating scale [NRS]) for 5 days, with the highest score for each cycle separately, and at least 2 days of moderate-to-severe pelvic pain (0–3 verbal rating scale [VRS]) on bleeding days and non-bleeding days. Subjects were required to have a BMI of ≥18 kg/m^2^. All enrolled participants were instructed to use non-hormonal, double-barrier contraception, such as condoms or a diaphragm, each combined with a spermicide.

Potential participants were excluded if they had liver enzyme anomalies, osteoporosis, or other metabolic bone disease or if they had taken specific medications like COC, GnRH agonists, or systemic glucocorticoids, within a specified wash-out period for each based on the time period these medications were expected to have a continued effect. A full list of inclusion and exclusion criteria is provided in Supplementary Table S1.

The full analysis set included 484 women, 160 taking 75 mg linzagolix, 162 taking 200 mg linzagolix with ABT, and 162 taking a drug-free pill (placebo). Demographic data are reported in Table 1.

**Table 1.** Baseline characteristics.

Characteristic	Placebo	Linzagolix 75 mg	Linzagolix 200 mg + ABT
	(N = 162)	(N = 160)	(N = 162)
Age (years), mean (SD)	34.9 (6.8)	35.1 (6.4)	34.6 (6.8)
Race			
White, n (%)	160 (98.8)	158 (98.8)	159 (98.1)
Black or African-American, n (%)	2 (1.2)	1 (0.6)	1 (0.6)
Asian, n (%)	0	1 (0.6)	0
Other, n (%)	0	0	0
BMI (kg/m^2^), mean (SD)	24.14 (4.44)	24.60 (5.23)	24.09 (5.17)
Time since first seeking medical diagnosis/treatment (years), mean (SD)	4.94 (4.51)	5.15 (4.38)	5.50 (4.74)
Pain scores			
Baseline overall pelvic pain VRS*, mean (SD)	1.90 (0.40)	1.87 (0.41)	1.92 (0.42)
Baseline non-menstrual pelvic pain VRS*, mean (SD)	1.78 (0.44)	1.73 (0.46)	1.80 (0.46)
Baseline dysmenorrhea VRS*, mean (SD)	2.29 (0.43)	2.25 (0.40)	2.29 (0.43)
Baseline overall pelvic pain NRS, mean (SD)	6.18 (1.51)	5.84 (1.65)	6.12 (1.60)
Baseline dyspareunia (VRS) mean (SD)	1.95 (0.87)	1.92 (0.86)	2.09 (0.84)
Baseline EHP-30 pain dimension, mean (SD)	51.77 (14.43)	51.46 (14.28)	52.37 (15.14)
Baseline analgesic use on bleeding days* (pill count/day), mean (SD)	1.65 (1.45)	1.88 (1.69)	2.01 (1.95)
Baseline analgesic use on non-bleeding days* (pill count/day), mean (SD)	0.78 (0.98)	1.00 (1.23)	1.08 (1.26)
Baseline dyschezia NRS, mean (SD)	4.46 (2.51)	3.73 (2.60)	4.07 (2.79)
Baseline Lipid levels			
LDL cholesterol (mg/dl), mean (SD)	114.3 (31.8)	117.4 (39.4)	115.4 (32.1)
Triglycerides (mg/dl), mean (SD)	91.9 (46.8)	92.0 (48.5)	90.0 (43.2)
Baseline BMD			
Femoral neck (g/cm^2^), mean (SD)		0.995 (0.141)	0.980 (0.144)
Lumbar spine (g/cm^2^), mean (SD)	1.214 (0.140)	1.197 (0.150)	1.169 (0.134)
Total hip (g/cm^2^), mean (SD)	1.031 (0.116)	1.026 (0.127)	1.015 (0.125)

* Based on the two selected screening menstrual cycles.

ABT: add-back therapy; BMD: bone mineral density; EHP-30: endometriosis health profile-30; LDL: low-density lipoprotein; NRS: numeric rating scale; VRS: verbal rating scale.

### Randomization and blinding

Patients were randomized according to a computer-generated randomization list prepared using Statistical Analysis System software version 9.4 (SAS Institute Inc., Cary, NC, USA) (Fig. 1). Prior to the start of the study, a randomization list and two treatment kit lists (one for linzagolix/placebo, one for ABT/placebo) were generated by a designated statistician from the sponsor or delegated to be transmitted to the assigned clinical packaging organization for labeling and to a fully integrated Interactive Web Response System (IWRS). The IWRS provided the kit numbers, which correspond to the linzagolix/placebo and ABT/placebo kit numbers. Subjects were randomized to one of three treatment groups in a 1:1:1 ratio. There was no stratification.

Placebo treatments were indistinguishable in appearance from active treatments. The allocation of treatment was concealed from subjects, investigators, and the trial operations team (including the sponsor).

### Primary and secondary endpoints

The primary objective was to demonstrate the efficacy and safety of linzagolix administered orally once daily for up to 3 months (12 weeks) at a dose of 75 mg alone or 200 mg with ABT, versus a placebo, in the management of moderate-to-severe endometriosis-associated pain in women with surgically confirmed endometriosis. The two co-primary efficacy endpoints were a clinically meaningful reduction of dysmenorrhea and non-menstrual pelvic pain over the last 28 days of randomized treatment up to Month 3, along with stable or decreased use of analgesics for endometriosis-associated pain. The clinically meaningful reduction thresholds for the primary analysis of dysmenorrhea and non-menstrual pelvic pain were determined on blinded Month 3 data using appropriate anchors and were defined as a reduction of at least 1.10 and 0.8 points on the VRS for dysmenorrhea and non-menstrual pelvic pain, respectively, as measured using an electronic diary (eDiary).

Further secondary endpoints were established to examine (at each study visit) any clinically meaningful reductions in dysmenorrhea and non-menstrual pelvic pain, as well as changes from baseline in the following outcomes: overall pelvic pain, mean worst pelvic pain, dyspareunia, dyschezia, general analgesic and opioid analgesic use for endometriosis-associated pain, ability to perform daily activities, Endometriosis Health Profile (EHP-30) dimensions (pain, control and powerlessness, emotional wellbeing, social support, self-image and modular sexual relationship questionnaire), and responses to the patient global impression of change (PGIC) and monthly patient global impression of severity (Mpgis) questionnaires. Additional secondary endpoints investigated the change from baseline to each visit as the number of days with uterine bleeding (including spotting) and assessed health/economy outcomes using the health-related productivity questionnaire (HRPQ).

Secondary efficacy objectives included evaluation of continued efficacy over the last 28 days of randomized treatment up to the Month 6 visit.

### Safety

Safety analyses were conducted using a safety analysis set. All safety assessments were summarized and listed, and the following endpoints were defined: (i) change from baseline to each scheduled assessment of BMD measured by DXA of the lumbar spine (L1-L4), femoral neck, and total hip; (ii) incidence and severity of treatment-emergent adverse events (TEAEs); (iii) time to the first post-treatment menses; (iv) changes in clinical laboratory analyses (hematology, biochemistry, coagulation parameters, hormones, lipids, and urinalysis) from baseline to each scheduled assessment; and (v) pathological alterations from baseline in the endometrium, as evidenced by histological findings from endometrial biopsies. Other safety endpoints were changes from baseline to each scheduled evaluation of any other safety parameter, including weight, vital signs, ECG, gynecological examinations, and endometrial thickness.

TEAEs were defined as adverse events starting on the day of or after the first dose of the study drug through to 30 days after its discontinuation or the Month 6 visit date. They also included any issue that was present at baseline but worsened in intensity or was subsequently considered drug-related by the investigator.

### Statistical analysis

Efficacy assessments were conducted using the full analysis set (FAS) and per protocol (PP) sets. Responder threshold analysis was performed for co-primary endpoints and ranked secondary endpoints using meaningful change thresholds (MCTs) were estimated from blinded data using anchor-based methods. MCTs thought to represent clinically meaningful reductions in pain are provided in parentheses for the relevant endpoints below.

Analysis of each co-primary endpoint was done using a logistic regression model, with the treatment group as the main effect (three values) and the baseline pain score as a covariate. Individual linzagolix versus placebo treatment group comparisons were made using the same logistic regression model. Estimated odds ratios (ORs) and 97.5% CIs for proportions of responders in linzagolix treatment groups versus the placebo were obtained, along with Bonferroni-corrected *P*-values. Estimates of the proportions of responders with 95% CIs were also acquired, based on the overall mean baseline pain score. In addition, sensitivity analyses using the Cochran–Mantel–Haenszel (CMH) test were applied to evaluate the null hypothesis of no treatment effect at Month 3 for each linzagolix group versus the placebo in terms of the proportions of subjects with a response for dysmenorrhea and non-menstrual pelvic pain. Odds ratios were estimated from the CMH test together with associated 97.5% CIs and corresponding Bonferroni-corrected *P*-values. Proportions per treatment arm were ascertained, together with exact Clopper–Pearson 95% CIs. Sensitivity analysis was also performed, which included any analgesic medication also taken by the responder for non-endometriosis-associated pain, based on the concomitant medication page in the electronic case report form (ECRF). The primary analysis model and descriptive statistics were repeated for each co-primary endpoint in the subgroups, including race, ethnicity, age, weight, BMI, baseline analgesic use and baseline dysmenorrhea (VRS), baseline non-menstrual pelvic pain (VRS) score, baseline dyspareunia (VRS) score, time since endometriosis diagnosis, and history of pregnancy.

Ranked secondary endpoints followed a fixed-sequence testing strategy within individual groups, to maintain the family-wise type I error rate. Indeed, comparisons for each linzagolix group versus the placebo for every ranked endpoint only reached a statistically significant difference if the raw *P*-value multiplied by two was ≤0.05 for that particular endpoint and all preceding endpoints for that particular dose versus the placebo. All secondary efficacy endpoints were summarized by descriptive statistics for each treatment group and timepoint, including summaries of change from baseline when applicable. As with the primary analysis, comparisons were made between individual linzagolix versus placebo treatment groups. Analyses of change from baseline in dysmenorrhea, non-menstrual pelvic pain, dyschezia, overall pelvic pain, EHP-30 pain dimensions, and dyspareunia were conducted using analysis of covariance (ANCOVA), with treatment group as the main effect (three values) and baseline pain scores as covariates.

Proportions of subjects reporting no analgesic use for endometriosis-associated pain, and those reporting no opiate use for endometriosis-associated pain during the 4-week period preceding Month 6 (last 28 days prior to and including the last treatment date) were analyzed by logistic regression. Data on analgesic use for endometriosis-associated pain and on concomitant medications were collected in the eDiary and the Ecrf, respectively, and evaluated for each treatment group (3 values).

All additional efficacy endpoints were collated by descriptive statistics for each treatment group and timepoint, including summaries of change from baseline where applicable. Each linzagolix group was compared to the placebo.

In general, between-group comparisons for continuous endpoints were evaluated by analysis of variance (ANOVA) or ANCOVA, and binary endpoints by logistic regression. Between-group comparisons for ordinal categorical data were analyzed using the CMH test or Koch’s method when there was also a baseline covariate. Further secondary endpoints used Bonferroni correction of *P*-values for comparisons of treatment groups at each visit. These analyses were not part of the fixed-sequence strategy implemented for co-primary and ranked secondary endpoints, and were not fully controlled for overall type I error rates.

For BMD analyses, calibrated values were used in summary tables where available (otherwise initial values were applied). BMD values and corresponding *Z*-scores were collated for each treatment group. Within-group percentage changes in BMD values from baseline were also collated, including two-sided 95% CIs. Changes from baseline were summarized as subjects falling in predefined percentage categories, such as no change or increase, 3% decrease, >3–5% decrease, >5–7% decrease, >7–8% decrease, and finally >8% decrease. Subgroup analyses by race, age, weight, and BMI were carried out for BMD, including descriptive summaries, percentage change category summaries, and ANCOVA.

## Results

### E2 levels

**Figure 2.**
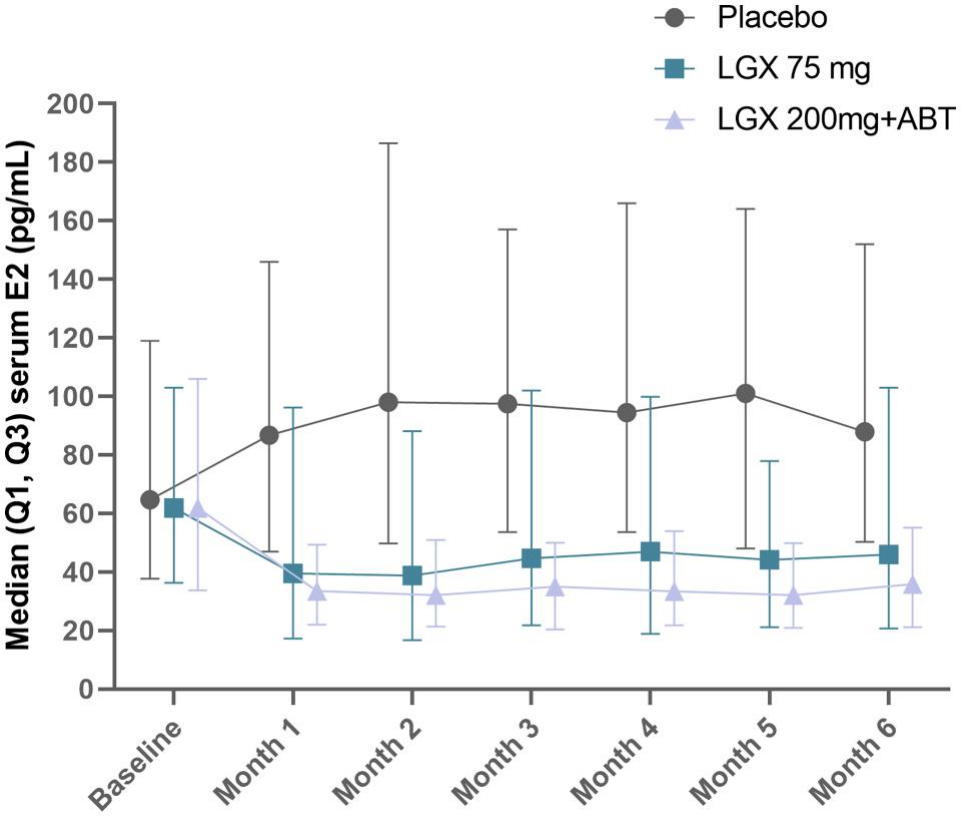
**Median estradiol levels during the early follicular phase in patients who received 75 mg linzagolix daily, those who received 200 mg linzagolix with ABT daily, and the placebo group.** ABT: add-back therapy; LGX: linzagolix; OR: odds ratio; PBO: placebo.

E2 levels were maintained within a therapeutic range (between 20 and 60 pg/ml), similar to that naturally observed during the early follicular phase, in patients who received 75 mg linzagolix daily as well as those who received 200 mg linzagolix with ABT daily (Fig. 2). The majority of subjects in the placebo group exhibited E2 levels of >60 pg/ml throughout the treatment period.

### Co-primary endpoints

**Figure 3.**
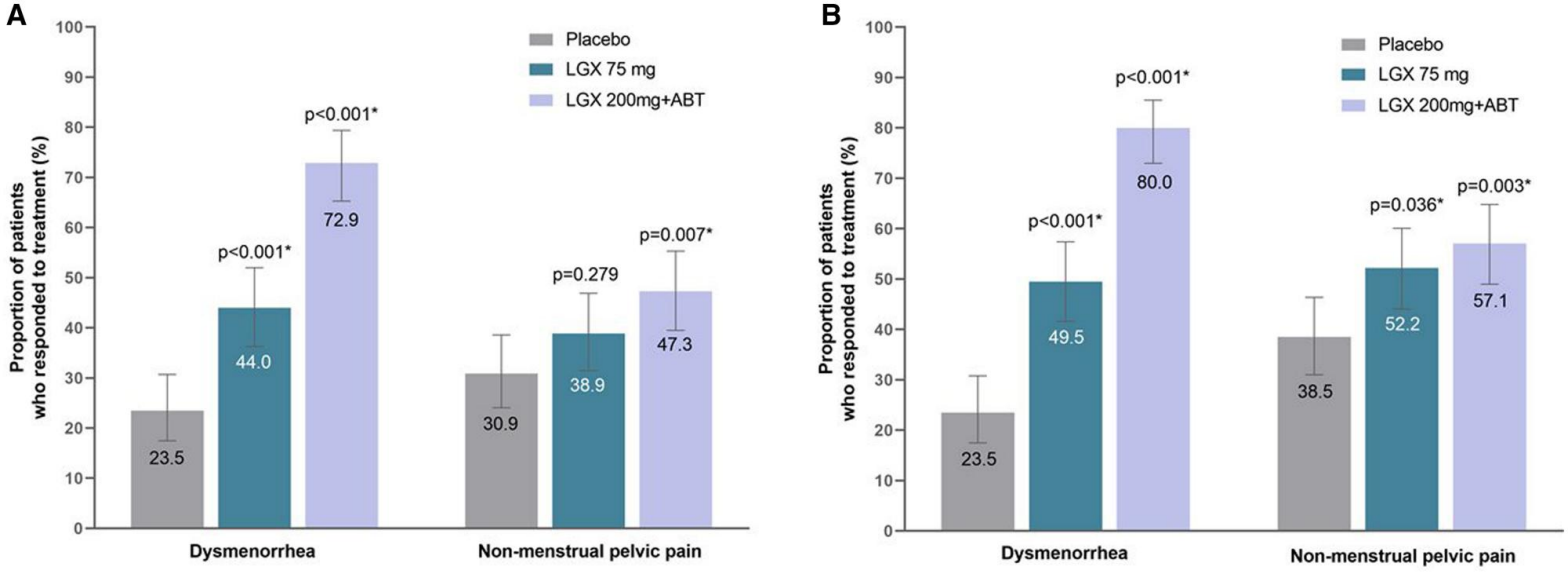
**Dysmenorrhea and non-menstrual pelvic pain in women treated by placebo, 75 mg linzagolix daily or 200 mg linzagolix plus ABT, at 3 and 6 months.** (**A**) Dysmenorrhea and non-menstrual pelvic pain at 3 months (mean ± 95% CI). Error bars represent 95% Cls. *Clinically significant value. **(B**) Dysmenorrhea and non-menstrual pelvic pain at 6 months (mean ± 95% CI). Error bars represent 95% Cls. *Clinically significant value. ABT: add-back therapy; LGX: linzagolix; OR: odds ratio; PBO: placebo.

The daily dose of 200 mg linzagolix with ABT met the primary efficacy objective, demonstrating clinically meaningful reductions (VRS) in dysmenorrhea and non-menstrual pelvic pain at 3 months, with stable or reduced use of analgesics. Based on logistic regression analysis, the estimated percentage of responders for dysmenorrhea (Fig. 3A) in the 200 mg linzagolix with ABT group was 72.9% (95% CI: 65.3, 79.4), but just 23.5% (95% CI: 17.5, 30.7) in the placebo group, with an OR of 8.80 (97.5% CI: 4.86, 15.91) and a Bonferroni-corrected *P*-value for treatment effect of <0.001. The proportion of responders for non-menstrual pelvic pain (Fig. 3A) in the 200 mg linzagolix with ABT group was 47.3% (95% CI: 39.5, 55.3) compared to 30.9% (95% CI: 24.1, 38.6) in the placebo group, with an OR of 2.01 (97.5% CI: 1.18, 3.42) and a Bonferroni-corrected *P*-value for treatment effect of 0.007.

The daily 75 mg linzagolix dose without ABT demonstrated a clinically meaningful and statistically significant reduction in dysmenorrhea versus placebo at 3 months. The proportion of responders for dysmenorrhea in the 75 mg linzagolix group was 44.0% (95% CI: 36.3, 52.0) compared to 23.5% (95% CI: 17.5, 30.7) in the placebo group, with an OR of 2.56 (97.5% CI: 1.46, 4.49) and a Bonferroni-corrected *P*-value for treatment effect of <0.001. Although the 75 mg dose showed a trend toward reduction in non-menstrual pelvic pain at 3 months versus the placebo, it was not statistically significant (*P* = 0.279 with an OR of 1.43).

### Ranked secondary endpoints

A fixed-sequence testing strategy was used within each treatment group to test ranked secondary endpoints. The 200 mg linzagolix with ABT group exhibited statistically significant differences compared to the placebo in both dysmenorrhea (*P* < 0.001) and non-menstrual pelvic pain (*P* < 0.001) at 6 months (Fig. 3B). The 75 mg linzagolix group also displayed significant differences in relation to placebo in both dysmenorrhea (*P* = 0.036) and non-menstrual pelvic pain (*P* = 0.003) at 6 months. This is further illustrated in Supplementary Fig. S1; the parallel and well-separated linear regression lines in these scatterplots perfectly demonstrate the effectiveness of linzagolix groups on dysmenorrhea. Despite certain outliers causing a crossover of linear regression lines toward the higher end of the baseline scores, the better efficacy of linzagolix groups is still evident for the non-menstrual pain endpoint.

There were statistically significant reductions (improvements) in the following ranked secondary endpoints at 6 months in the 200 mg linzagolix with ABT group compared to the placebo: dyschezia, overall pelvic pain and the ability to participate in daily activities gauged using pain dimensions listed in the EHP-30 (Table 2).

**Table 2.** Primary and secondary efficacy endpoints at 3 and 6 months.

	Placebo	Linzagolix 75 mg			Linzagolix 200 mg + ABT		
	(N = 162)	(N = 160)			(N = 162)		
	Proportion of responders (95% CI)	Proportion of responders (95% CI)		***P*-value** ^1^	Proportion of responders (95% CI)		***P*-value** ^1^
**Dysmenorrhea at Month 3**	23.5 (17.5; 30.7)	44.0 (36.3; 52.0)		<0.001	72.9 (65.3; 79.4)		<0.001
**Non-menstrual pelvic pain at Month 3**	30.9 (24.1; 38.6)	38.9 (31.5; 46.9)		0.279	47.3 (39.5; 55.3)		0.007

	**LSM (95% CI)**	**LSM (95% CI)**	**Diff with PBO (97.5% CI)**	***P*-value** ^1^	**LSM (95% CI)**	**Diff with PBO (97.5% CI)**	***P*-value** ^1^

**Change from baseline in dysmenorrhea (VRS) after 6 months**	−0.66 (−0.79; −0.53)	−1.10 (−1.23; −0.97)	−0.44 (−0.65; −0.23)	<0.001	−1.83 (−1.96; −1.70)	−1.17 (−1.38; −0.97)	<0.001
**Change from baseline in NMPP (VRS) after 6 months**	−0.66 (−0.77; −0.56)	−0.84 (−0.95; −0.73)	−0.17 (−0.35; 0.00)	0.048	−0.92 (−1.03; −0.82)	−0.26 (−0.43; −0.09)	0.002
**Change from baseline in dyschezia (NRS)**	−1.41 (−1.71; −1.12)	−1.98 (−2.28; −1.69)	−0.57 (−1.05; −0.09)	0.015	−1.99 (−2.29; −1.70)	−0.58 (−1.05; −0.11)	0.012
**Change from baseline in overall pelvic pain (NRS) at 6 months**	−2.19 (−2.55; −1.84)	−2.84 (−3.20; −2.48)	−0.65 (−1.23; −0.07)	0.024	−3.39 (−3.74; −3.03)	−1.19 (−1.77; −0.62)	<0.001
**Change from baseline in EHP-30 Pain dimension: analysis of covariance (FAS) at 6 months**	−19.47 (−22.66; −16.28)	−27.37 (−30.50; −24.25)	−7.90 (−13.01; −2.79)	0.001	−35.60 (−38.73; −32.48)	−16.13 (−21.24; −11.02)	<0.001
**Change from baseline in dyspareunia (VRS) at 6 months: analysis of covariance (FAS)**	−0.82 (−0.97; −0.66)	−1.04 (−1.21; −0.88)	−0.23 (−0.49; 0.03)	0.100	−1.01 (−1.18; −0.85)	−0.20 (−0.46; 0.07)	0.184

	**% of responders (95%CI)**	**% of responders (95%CI)**	**OR** ^2^ **(97.5% CI)**	***P*-value** ^1^	**% of responders (95%CI)**	**OR** ^2^ **(97.5% CI)**	***P*-value** ^1^

**No analgesic use for EAP during the preceding 4-week period at Month 6**	13.2 (8.9; 19.2)	30.9 (23.8; 39.1)	2.95 (1.58; 5.50)	<0.001	44.5 (36.3; 52.9)	5.27 (2.83; 9.82)	<0.001
**No opiate use for EAP during the preceding 4-week period at Month 6**	97.0 (92.5; 98.9)	93.8 (88.5; 96.8)	0.47 (0.12; 1.82)	0.420	97.0 (92.7; 98.8)	0.99 (0.22; 4.51)	1.000

ABT: add-back therapy; EAP: endometriosis-associated pain; EHP-30: Endometriosis Health Profile-30; FAS: full analysis set; LSM: least square mean; NMPP: non-menstrual pelvic pain; NRS: numeric rating scale; OR: odds ratio; PBO: placebo; VRS: verbal rating scale.

Scores were computed as mean of daily assessments on the last 28 days prior to Month 6 or discontinuation.

Analysis of covariance with change from baseline as response variable, and baseline value, treatment as covariates.

EHP-30 Pain Score at Month 6 or discontinuation. EHP-30 Pain Score was computed as the sum of each pain question score/(number of items × 4) × 100 and ranges from 0 (best possible health status) to 100 (worst possible health status).

For dyspareunia, subjects not sexually active for other reasons than endometriosis have missing value.

Analgesic and opiate use for EAP as collected in eDiary and Concomitant Medication page on the last 28 days prior to Month 6 or discontinuation.

1 Bonferroni-corrected p-value. A fixed-sequence testing strategy was used to account for multiplicity. The LGX 200 mg + ABT group demonstrated statistical significant differences for both co-primary endpoints. The LGX 75 mg group was not found to be statistically significantly compared to placebo for the NMPP co-primary endpoint.

2 Odds ratios and 97.5% CI estimated with logistic regression model with no analgesic use/no opiate use in the last 28 days on treatment prior to Month 6 as response variable, treatment group as the main effect and including the baseline analgesic use/opiate use as a covariate.

**Table 3.** Adverse events.

	Placebo	Linzagolix 75 mg	Linzagolix 200 mg + ABT
	(N = 162)	(N = 160)	(N = 162)
	n (%)	n (%)	n (%)
**Any TEAE**	76 (46.9)	75 (46.9)	92 (56.8)
**Severe TEAE**	2 (1.2)	5 (3.1)	3 (1.9)
**Serious TEAE**	0 (0)	1 (0.6)	2 (1.2)
**TEAE leading to permanent discontinuation of treatment**	4 (2.5)	9 (5.6)	5 (3.1)
**TEAEs occurring in more than 5% of subjects in any linzagolix group**			
**Headache**	13 (8.0)	13 (8.1)	17 (10.5)
**Hot flush**	4 (2.5)	12 (7.5)	11 (6.8)
**Fatigue**	4 (2.5)	6 (3.8)	11 (6.8)

ABT: add-back therapy; LGX: linzagolix; TEAE: treatment-emergent adverse event.

For subjects not entering the follow-up or the extension period, AE are included in this summary up to 30 days after end of treatment. Only one subject (407016) not entering the follow-up or extension period reported 2 AE more than 30 days after end of treatment.

Reductions in these ranked secondary endpoints were also observed at 6 months with the 75 mg linzagolix dose. These improvements were typically lower in magnitude than those observed with 200 mg linzagolix with ABT, except for dyschezia, where the treatment effect was similar with both dose regimens (Table 2).

### Additional secondary endpoints

Response to treatment was rapid. Reductions in dysmenorrhea and non-menstrual pelvic pain compared to the placebo were observed with both doses after 1 and 2 months of treatment. At Month 1, proportions of responders for dysmenorrhea were markedly higher than with the placebo for both linzagolix groups, with ORs of 3.79 (97.5% CI: 1.77, 8.08; *P* < 0.001) and 3.90 (97.5% CI: 1.84, 8.27; *P* < 0.001) for the 75 mg linzagolix and 200 mg linzagolix with ABT groups, respectively. At Month 2, the proportion of responders for non-menstrual pelvic pain was higher than in the placebo group for 200 mg linzagolix with ABT, with an OR of 2.36 (97.5% CI: 1.34, 4.16; *P* = 0.001), but not for 75 mg linzagolix with an OR of 1.72 (97.5% CI: 0.97, 3.07; *P* = 0.068).

A rapid response to treatment was also observed in terms of subject-reported numbers of days with moderate-to-severe pelvic pain during the preceding 4 weeks. In both linzagolix groups, the estimated number of days with moderate-to-severe pelvic pain fell from a mean (SD) of 18.5 (6.3) days at baseline to ∼12 days (95% CI ranges from 10.83 to 13.15) at Month 1. Further improvements were noted throughout the treatment period, with estimated numbers of days with moderate-to-severe pelvic pain at Month 6 of 7.28 (CI 5.86; 9.04) and 4.75 (CI 3.81; 5.92) days in the 75 mg linzagolix and 200 mg linzagolix with ABT groups, respectively. Accordingly, estimated mean numbers of pelvic pain-free days (during the preceding 4 weeks) gradually rose throughout the treatment period, with estimated numbers of pelvic pain-free days at Month 6 climbing from overall mean (SD) baseline values of 1.2 (2.7) to 8.10 days (95% CI: 6.05, 10.85) in the 75 mg linzagolix group and 8.81 days (95% CI: 6.60, 11.75) in the 200 mg linzagolix with ABT group.

Reduction in pelvic pain was associated with an observed drop in the use of analgesics for endometriosis-associated pain. Actual values and changes from baseline in the number of days with analgesic use recorded in the eDiary were summarized for the last 28 days prior to each visit. Subjects in both linzagolix groups achieved marked reductions in the number of days with analgesic use throughout the treatment period starting at Month 1, with further drops observed until Month 6. At Month 1, the estimated number of days with analgesic use for endometriosis-associated pain was cut by half (from an overall mean (SD) baseline of 12.4 (8.0) days to <6 days) in both groups. At Month 6, the estimated number of days was 3.73 for 75 mg linzagolix (95% CI: 2.96, 4.71; *P* = 0.015) and 2.35 for 200 mg linzagolix with ABT (95% CI: 1.85, 2.98; *P* < 0.001).

Subjects in the 200 mg linzagolix with ABT group achieved marked improvements in mean daily dyschezia scores as early as Month 2, showing a difference with the placebo of just −0.41 (97.5% CI: −0.80, −0.02; *P* = 0.038), maintained at 6 months −0.58 (97.5% CI: −1.05, −0.11; *P* = 0.012). Similarly, subjects in the 75 mg linzagolix group also saw improvements in mean daily dyschezia scores, differing from the placebo by just −0.35 (97.5% CI: −0.74, 0.04; *P* = 0.092) and maintaining a significant reduction at 6 months −0.57 (97.5% CI: −1.05, −0.09; *P* = 0.015). Small, non-significant, improvements in dyspareunia scores were observed in both linzagolix groups compared to the placebo.

The number of bleeding days (including spotting) decreased in both linzagolix groups by Month 2. By Month 6, the estimated number of days with uterine bleeding including spotting had fallen from an overall baseline mean (SD) of 6.6 (2.4) to 4.47 (95% CI: 3.74, 5.33) days in the 75 mg linzagolix group and 2.26 (95% CI: 1.87, 2.74) in the 200 mg linzagolix with ABT group. Improvements in endometriosis pain resulted in enhanced quality of life, as indicated by the ability to accomplish daily activities. Indeed, improved ability to perform daily activities, as reported in the eDiary, was observed in both linzagolix groups, and women in both groups recorded considerably better daily activity scores.

Quality of life was evaluated using the EHP-30 questionnaire. Interference of pain and daily functioning, assessed by the EHP-30 pain domain, improved significantly in both treated groups compared to the placebo, with an estimated mean change from baseline of −27.37 (95% CI: −30.50, −24.25) for the 75 mg linzagolix group and −35.60 (95% CI: −38.73, −32.48) for the 200 mg linzagolix with ABT group. By Month 6, significant improvements (total score reductions) were observed in all five dimensions (pain, control and powerlessness, emotional wellbeing, social support, and self-image) in the 200 mg linzagolix with ABT group, and in all but one dimension (social support) in the 75 mg linzagolix group compared to the placebo.

Subject-perceived impression of change, as determined by responses to the PGIC, and severity, as gauged by responses to the PGIS, also showed enhanced scores with treatment. In the 200 mg linzagolix with ABT group, better PGIC questionnaire scores were observed from Month 1 and at each visit throughout the treatment period, compared with the placebo. Subjects in the 75 mg linzagolix group also had improved scores on the PGIC questionnaire at all visits except Months 3 and 4. At Month 6, the OR for the probability of higher response categories versus the placebo was 0.56 (97.5% CI: 0.34, 0.89; *P* = 0.011) in the 75 mg linzagolix group and 0.18 (97.5% CI: 0.11, 0.30; *P* < 0.001) in the 200 mg linzagolix with ABT group. By Month 6, a higher proportion of subjects in the linzagolix groups (75 mg: 54.0%; 200 mg with ABT: 80.7%) considered their endometriosis symptoms either very much improved or much improved compared to the placebo (38.4%).

### Safety

The median duration of therapy was 24 weeks for each treatment group, with at least 50% of subjects remaining on therapy for between 23.6 and 24.2 weeks. Overall, linzagolix appeared to be well tolerated. The percentage of subjects reporting one or more TEAEs was the same in the placebo and 75 mg linzagolix groups (46.9% in both) and slightly higher in the 200 mg linzagolix with ABT group (56.8%) during the 6-month treatment period (Table 3). Most TEAEs (around 98%) were mild or moderate in intensity. The incidence of severe TEAEs was comparable between the placebo (1.2%) and linzagolix groups (75 mg: 3.1%; 200 mg with ABT: 1.9%).

The most commonly reported TEAEs were headaches, which were encountered at a similar rate in the three groups (placebo: 8.0%; 75 mg: 8.1%; 200 mg with ABT: 10.5%). Hot flushes occurred considerably more frequently in the linzagolix groups (75 mg: 7.5%; 200 mg with ABT: 6.8%) than in the placebo group (2.5%). Fatigue was reported at a similar rate in the placebo (2.5%) and 75 mg linzagolix (3.8%) groups, but slightly higher in the 200 mg linzagolix with ABT group (6.8%). There were no fatal TEAEs and no suicide-related TEAEs reported in the study.

**Figure 4.**
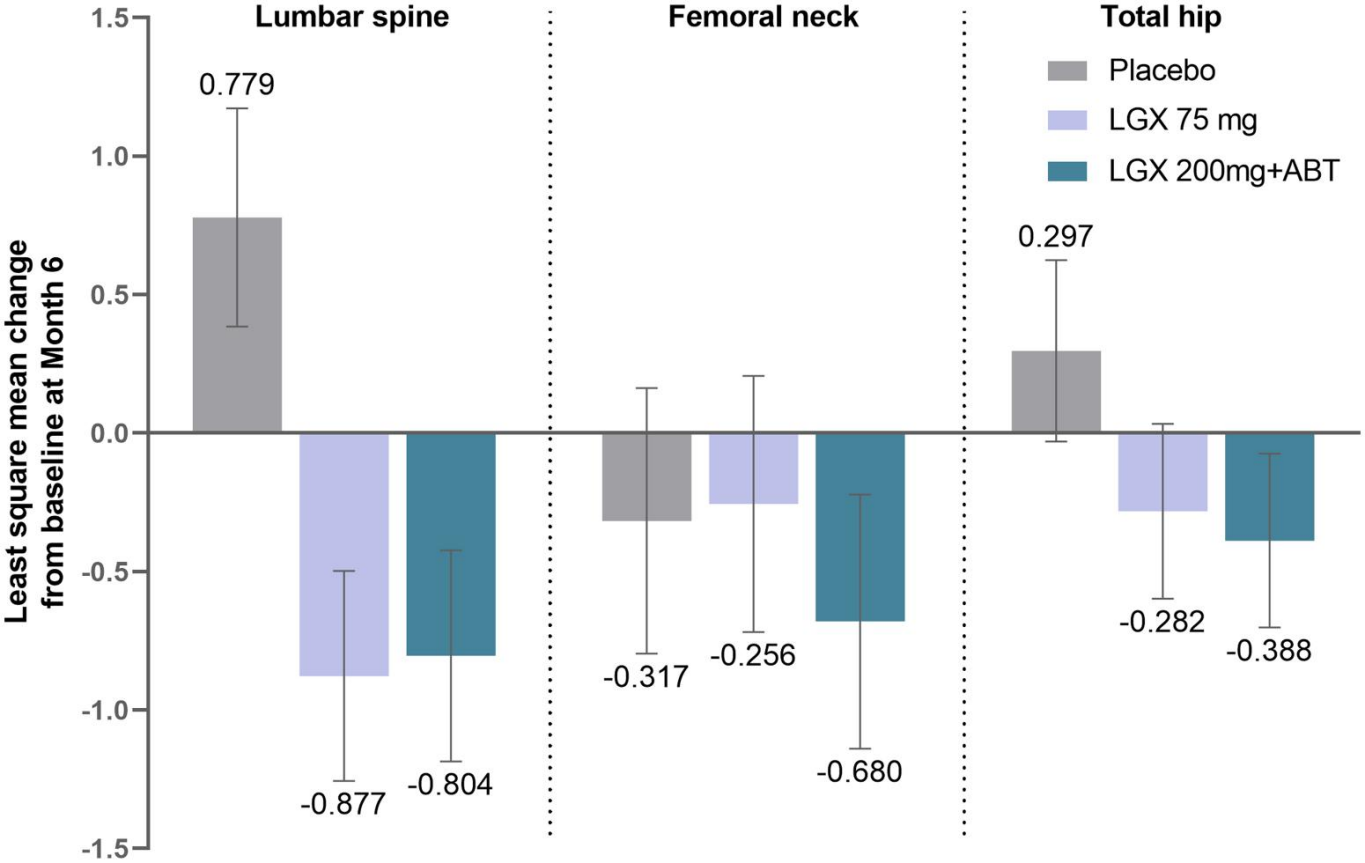
**Changes in bone mineral density (BMD) from baseline at 6 months.** The mean percentage change from baseline was <1% in all three groups (placebo, 75 mg and 200 mg with ABT) in the lumbar spine, femoral neck, and total hip. Error bars represent 95% Cls. ABT: add-back therapy; diff: difference; LGX: linzagolix; LSM: least square mean; OR: odds ratio; PBO: placebo.

Changes in BMD were minimal. Figure 4 illustrates the least square mean change (LSM) from baseline at 6 months. The mean percentage change from baseline was <1% in all three groups (placebo, 75 mg and 200 mg with ABT) in the lumbar spine, femoral neck, and total hip. In the linzagolix groups, most subjects experienced either an increase, no change, or decrease of <3% in BMD at Month 6 in the lumbar spine (84.5%), total hip (94.3%) and femoral neck (88.2%). Reductions of more than 8% were observed in all treatment groups: one subject in the placebo group (femoral neck), one subject in the 75 mg group (lumbar spine), and four subjects in the 200 mg with ABT group (3 femoral neck and 1 total hip).

Concerning *Z* scores at Month 6, changes from baseline were comparable between all three groups in all measured bone sites. Subgroup analyses revealed that subjects aged over 35 years and those weighing <63 kg or with a BMI under 23 kg/m^2^ appeared to suffer more bone loss in all bone sites.

Twelve subjects showed elevated liver enzyme parameters during treatment, which were considered clinically significant by the investigator: three subjects (1.9%) in the placebo group, six subjects (3.2%) in the 75 mg linzagolix group, and three subjects (1.9%) in the 200 mg linzagolix with ABT group, including one subject who had alanine transaminase (ALT)/aspartate aminotransferase (AST) levels of more than three times the upper limit of normal (ULN). Elevated liver enzymes were observed only over a very short period and were reversible during treatment. One subject had an isolated AST increase with concomitant creatine kinase (CK) increase due to excessive physical training which was rapidly reversed*.* In addition, one subject in the 200 mg linzagolix with ABT group exhibited gamma-glutamyl transpeptidase (GGT) elevation of ≥3× the ULN. All other transaminase increases were <3× the ULN.

In terms of lipids, two subjects had elevated triglyceride levels, which were considered clinically significant by the investigators. One subject in the placebo group displayed high triglyceride levels at Month 4 (428 mg/dl; Grade 2), Month 5 (392 mg/dl; Grade 2) and Month 6 (327 mg/dl; Grade 2). This subject already showed Grade 1 elevation (187 mg/dl) at baseline, but her values normalized when she was switched to 75 mg linzagolix during the extension study. Another subject (in the 75 mg linzagolix group) exhibited a single increased triglyceride level at Month 3 (637 mg/dl; Grade 3) which returned to normal values under continued treatment.

Treatment discontinuation rates due to TEAEs were comparable between the placebo (2.5%) and 200 mg linzagolix with ABT group (3.1%), and only slightly higher in the 75 mg linzagolix group (5.6%).

### Post-treatment evolution: follow-up during the drug-free period

In 51 patients who did not continue into the extension study, menses resumed after cessation of therapy in both linzagolix groups. Estimated time to recovery of menses was 4–5 weeks thanks to rapid recovery of ovarian function (assessed by blood tests). During the drug-free follow-up period, efficacy was generally briefly maintained for the first 3 months and decreased after a longer time without treatment. Due to the small number of patients in the follow-up set which resulted in high variability, the results should be interpreted with caution. At 1 month of follow-up, the proportions of dysmenorrhea responders were higher in the 75 mg linzagolix (25.0%) and 200 mg linzagolix with ABT (45.0%) groups than in the placebo group (10.0%). At 6 months of post-treatment follow-up, the proportions of dysmenorrhea responders fell in both linzagolix groups (75 mg: 14.3%; 200 mg with ABT: 28.6%). Rates of non-menstrual pelvic pain responders did not indicate any particular pattern and once again demonstrated high variability between the different timepoints.

## Discussion

Endometriosis is a chronic, progressive, inﬂammatory and estrogen-dependent disease that requires a life-long management plan (Lousse *et al.*, 2012; Patel *et al.*, 2018; Donnez and Dolmans, 2021a,b; Taylor *et al.*, 2021; Horne and Missmer, 2022). Costs linked to endometriosis are high (Soliman *et al.*, 2016, 2017, 2018), so it is essential to promote continued research into medical alternatives that enhance quality of life in all affected women.

First-line therapies include combined COC or progestogen treatment (Vercellini *et al.*, 2016, 2018a,b; Casper, 2017), which are effective in two-thirds of women with endometriosis-related pain. The notion of progesterone resistance explains why one-third of patients do not respond to these drugs (Flores *et al.*, 2018; Reis *et al.*, 2020; Donnez and Dolmans, 2021a; Bulun *et al.*, 2023).

Use of GnRH agonists without ABT, considered a second-line approach, is limited to 6 months because they are associated with serious adverse events due to complete suppression of estrogen production (Donnez *et al.*, 2002; Olive, 2008; Becker *et al.*, 2022). Combining GnRH agonists with various add-back therapies greatly limit the adverse events caused by the complete suppression of estrogen production.

Several studies have published findings from clinical trials on three potentially effective oral GnRH antagonists (Taylor *et al.*, 2017; Donnez *et al.*, 2020; Donnez and Dolmans, 2021b; Giudice *et al.*, 2022).

The advantages of a GnRH antagonist plus ABT compared to a GnRH agonist plus ABT are: (i) oral administration; (ii) no flare-up effects; (iii) the dose-dependent decrease in ovarian steroid secretion; and (iv) rapid reversibility (Donnez *et al.*, 2020; Donnez and Dolmans, 2021a,b).

As detailed in this report, the Phase 3 EDELWEISS 3 trial, which involved women with moderate-to-severe endometriosis-related pain, evaluated two doses of linzagolix: a once-daily 200 mg dose in combination with hormonal add-back therapy (ABT) and a 75 mg dose without ABT. Doses were selected on the basis of results from the dose-finding EDELWEISS 1 study (Donnez *et al.*, 2020). In the EDELWEISS 3 study, the 200 mg plus ABT regimen met the co-primary objectives in terms of efficacy, showing a significant decline in dysmenorrhea and non-menstrual pelvic pain by 3 months of therapy.

Indeed, a significantly larger percentage of women treated with this combination showed a response to the treatment compared to those who received a placebo. The 75 mg regimen without hormonal add-back therapy (ABT) demonstrated a statistically significant enhancement in alleviating dysmenorrhea compared to the placebo after 3 months. However, while this dose alleviated non-menstrual pelvic pain to some extent by 3 months, the difference was not statistically significant in comparison to the placebo, therefore it did not meet the co-primary efficacy objective.

By 6 months, both dysmenorrhea and non-menstrual pelvic pain were significantly improved with both doses. Among women treated with linzagolix 200 mg plus ABT, the percentage of women with a reduction in dysmenorrhea (72.9̶–80% at 3 and 6 months, respectively) was higher than those with a reduction in non-menstrual pelvic pain (47.3–57.1%). This is consistent with the mechanism of action of this combination, which significantly shortens the number of bleeding days. A similar observation was made by Giudice *et al.* (2022) in women given relugolix combination therapy. Indeed, mechanisms governing non-menstrual pelvic pain are probably multifactorial, such as chronic peritoneal inflammation, pelvic adhesions, generation of myofascial trigger points, and central sensitization, and are probably less responsive to decreased levels of E2 than dysmenorrhea (Aredo *et al.*, 2017). A further argument supporting this hypothesis emerges from comparing responder rates in the group of women treated with 75 mg alone. Higher responder rates were observed for dysmenorrhea than for non-menstrual pelvic pain, but the responder rate for dysmenorrhea, while significant, was lower than that observed in the 200 mg plus ABT group, where the number of bleeding days was reduced.

Clinically meaningful improvements were observed in other crucial secondary endpoints, specifically dyschezia, overall pelvic pain, and the capacity to perform normal daily activities, as measured by the EHP-30 pain dimension scale. Decreased analgesic and opioid use was also an indicator of efficacy on pain. From an overall mean baseline of 12.4 days, the estimated number of days with analgesic use was cut by half (to <6 days) at Month 1. At Month 6, the estimated number of days was 3.73 for 75 mg linzagolix and 2.35 for 200 mg linzagolix with ABT, representing a highly clinically relevant finding.

We show here that the reduction in dyspareunia in patients treated with linzagolix was not significant. Taylor *et al.* (2017) observed similar findings with elagolix. Indeed, a significant reduction in dyspareunia was only observed in the 200 mg elagolix group (twice a day) which is a very high dose that provokes significant bone mineral density (BMD) loss. Even in the high-dose arms of the study, the response rate was low (∼60%). When elagolix is given at a low dose of 150 mg, the decrease is not significant. The elagolix trials were larger and had more power to detect small differences. The lack of significant improvement in dyspareunia with linzagolix is likely a power issue. Only a subset of patients had dyspareunia which also limits the possibility of demonstrating an effect in smaller populations. Moreover, assessment of change in the case of dyspareunia is difficult, as it depends on sexual activity that is libido-dependent and varies from patient to patient.

All the significant changes in primary and secondary endpoints were consistent with rapid and differential suppression of serum estradiol levels, which nevertheless remained in the so-called optimal zone (between 20 and 60 pg/ml) most of the time.

The incidence of serious and non-serious side effects was similar across both linzagolix groups and the placebo group, proving good tolerability. There were no fatal issues or suicide-related TEAEs in the study.

It should be noted that BMD loss was not clinically meaningful (<1% from baseline for the lumbar spine, femoral neck, and total hip at 24 weeks). Changes in *Z* scores from baseline at Month 6 were comparable between all three groups in all measured bone sites. In the femoral neck, BMD loss was lower in the 75 mg group than in the placebo group. This strongly suggests that a daily dose of 75 mg could be administered without the need for hormonal add-back therapy.

Rates of hot ﬂushes and headaches were similar in both the treated groups and the placebo group. These data, along with those published on women with uterine fibroids treated with linzagolix (Donnez *et al.*, 2022) appear to confirm that an adequate estradiol range according to the threshold hypothesis (the so-called optimal zone) can address endometriosis symptoms while minimizing the negative effects of E2 deprivation.

Very importantly, the occurrence of elevated liver enzymes and lipid anomalies was very rare, observed only over a very short period, and reversible during treatment. These observed liver enzyme elevations were comparable with what was reported for other GnRH antagonists. No significant toxic effects (on the liver) have been reported in any studies published so far (Taylor *et al.*, 2017; Surrey *et al.*, 2018; Giudice *et al.*, 2022). This indicates that there is no need for close monitoring during treatment.

Three pregnancies were reported during the study, despite patients being required to use non-hormonal contraception: one in the placebo group (the women delivered a healthy baby) and two in the 75 mg group (known to not block ovarian function completely). In the two patients in the 75 mg group, one underwent an induced abortion and the second was lost to follow-up. We, therefore, suggest giving the 75 mg dose to adolescents and women without sexual activity or using a mechanical contraceptive method.

No pregnancies were reported in subjects given 200 mg linzagolix with ABT, which is well known to induce full suppression of ovarian activity and anovulation (Donnez *et al.*, 2022) after at least two weeks of therapy.

The strengths of this study are that it was a multinational, multi-centered, randomized, placebo-controlled trial in patients with surgically corroborated endometriosis and confirmed moderate-to-severe endometriosis pain at baseline. The study compared two different doses of linzagolix with a placebo. Assessments of pain were based primarily on patient-reported outcomes recorded daily in eDiaries using a verbal rating scale (VRS) for endometriosis-associated pain. Safety assessments included a controlled evaluation of BMD loss at 12 and 24 weeks. Finally, dose-dependent suppression and rapid reversibility of effects due to their short half-life are crucial issues for women of reproductive age, allowing clinicians to select the optimal dose: 75 mg alone or 200 mg plus ABT.

Linzagolix may possess an advantage regarding patient compliance as it can be administered as a single dose at any time during the day, without the risk of reduced and/or variable exposure to the active substance (Pohl *et al.*, 2022; Tezuka *et al.*, 2022).

The present study demonstrates that high doses of linzagolix plus ABT are effective (similar to relugolix plus ABT), but also that low doses without ABT can significantly alleviate both dysmenorrhea and non-menstrual pelvic pain (NMPP) at 6 months (similar to elagolix at a low dose of 150 mg). Compared to elagolix, linzagolix has a longer half-life, which may provide better and more consistent efficacy for the management of endometriosis-associated pain. In addition, this offers the possibility of treatment for women in whom combined oral contraceptives (COCs) or progesterone (OP) are contraindicated or who are reluctant to take it.

This study also has limitations. Data from comparative studies with estro-progestogens or progestogens would indeed be beneficial to determine whether GnRH antagonists offer significant advantages over traditional first-line medications. Trials examining their effects on pain symptoms, quality of life, side effects, tolerability, and treatment adherence should be undertaken. In the event of endometriosis-related symptoms, we strongly endorse the use of first-line therapy (combined oral contraceptives or progestogens). However, if these treatments fail, GnRH antagonists work through a different mechanism, estrogen deprivation, and are typically effective, even in those with progesterone resistance. Further, progestins have common side effects that include altered mood and bloating. Nevertheless, future studies should confirm the legitimacy of this approach, bearing in mind that the costs of any long-term medical treatment need to be carefully balanced (Donnez and Dolmans, 2021a,b). As mentioned by Vercellini *et al.* (2018a,b, 2019), further studies should evaluate if the magnitude of difference in efficacy of GnRH antagonists versus COC or progestogens is worth the substantial extra cost associated with use of GnRH antagonists.

The cost-effectiveness and the additional benefits of GnRH antagonists in comparison to traditional first-line therapies need to be evaluated and thoroughly examined, as well as their effectiveness, in women who are poor responders because of the widely recognized occurrence of progesterone resistance.

Since the treatment duration was only 6 months, the present study could not address efficacy and safety beyond this point. Longer-term efficacy and outcomes will be the subject of future reports, but preliminary data support the maintenance of efficacy and safety at 52 weeks in women entering the extension study.

### The place of GnRH antagonist in a long-term strategy

Regarding the role of GnRH antagonists in a long-term management plan, Vercellini *et al.* (2018a) envisioned a therapeutic pyramid, starting with a wide base comprising users of first-line medications (combined oral contraceptives: COC), narrowing progressively to users of second-line drugs (progestins), and becoming even more slender for patients on third-line therapies (GnRH agonists/antagonists), with a small pinnacle representing patients undergoing surgery. It is well acknowledged in clinical practice that patients may quickly lose confidence when various drugs are repeatedly employed with inadequate outcomes. We unequivocally endorse the use of first-line therapy (COC or progestins) yet stress the importance of offering an alternative to the 33% of women who are progesterone-resistant (Donnez and Dolmans, 2021a,b).

In conclusion, a combination of 200 mg linzagolix, estradiol, and norethisterone acetate was found to significantly reduce endometriosis-associated pain and improve quality of life, while minimizing risks of bone loss and vasomotor symptoms thanks to the add-back therapy. A daily dose of 75 mg linzagolix provided significant relief from dysmenorrhea at 3 months and both dysmenorrhea and non-menstrual pelvic pain at 6 months, with minimal BMD loss due to only partial suppression of serum estradiol. These characteristics suggest that lower doses of linzagolix could be suitable for chronic treatment of endometriosis-associated pain without the need for concomitant hormonal add-back therapy, and if confirmed by further research, would offer a viable option for women who do not wish to have ABT or in whom it is contraindicated.

Costs associated with endometriosis are estimated to be $80 billion annually (Soliman *et al.*, 2018), underscoring the urgent need to promote and encourage research, explore innovative treatment options, and enhance women’s access to quality care. Further investigations are required, including evaluations of efficacy and safety in real-world populations, potential applications of add-back therapy (ABT), and comparisons with combined oral contraceptives (COC) and progestins. It is imperative to identify and address the unmet needs in endometriosis care, and actions must be taken sooner rather than later.

Appropriate counseling of patients is fundamentally important. Healthcare providers are responsible for providing a comprehensive overview of the efficacy and side effects of all available therapies. The ideal treatment should then be customized for each individual woman based on their most troublesome symptoms (pain or infertility) and the phenotype of their disease.

## Supplementary Material

deae076_Supplementary_Figure_S1

deae076_Supplementary_Table_S1

## Data Availability

Appropriately de-identified patient-level datasets and supporting documents may be shared following the attainment of applicable marketing approvals and consistent with criteria established by Theramex and/or industry best practices to maintain the privacy of study participants. For more information, please contact mitra.boolell@theramex.com.
