# Supplementary material for: Linzagolix therapy versus a placebo in patients with endometriosis-associated pain: a prospective, randomized, double-blind, Phase 3 study (EDELWEISS 3)
Source: Hum Reprod. 2024 Apr 22;39(6):1208–21. doi: 10.1093/humrep/deae076 (PMC11144970; doi:10.1093/humrep/deae076)
Supplement: deae076_Supplementary_Figure_S1 [file deae076_supplementary_figure_s1.pdf]

OXFORD

human reproduction

Human Reproduction, 2024, 1–1

https://doi.org/10.1093/humrep/deae076

Supplementary Data

A

Change from Baseline to Month 6 for dysmenorrhea (VRS) – Full analysis set.

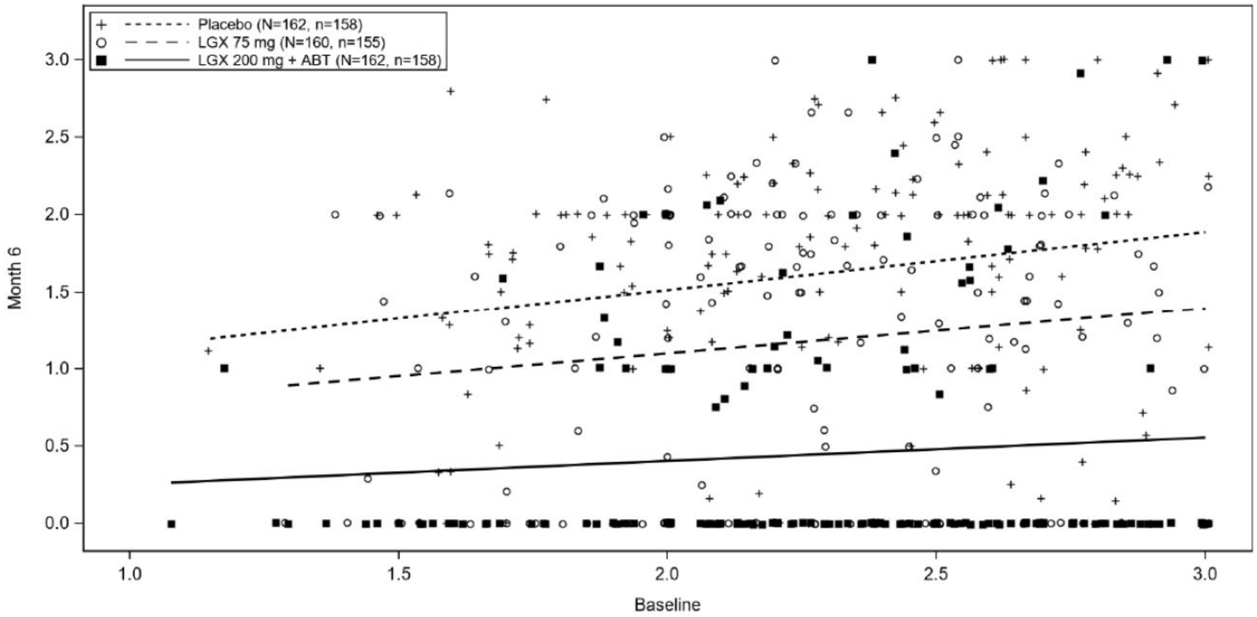

LGX: LINZAGOLIX; ABT: Add-Back Therapy; VRS: Verbal Rating Scale.

B

Change from Baseline to Month 6 for non-menstrual pelvic pain (VRS) – Full analysis set.

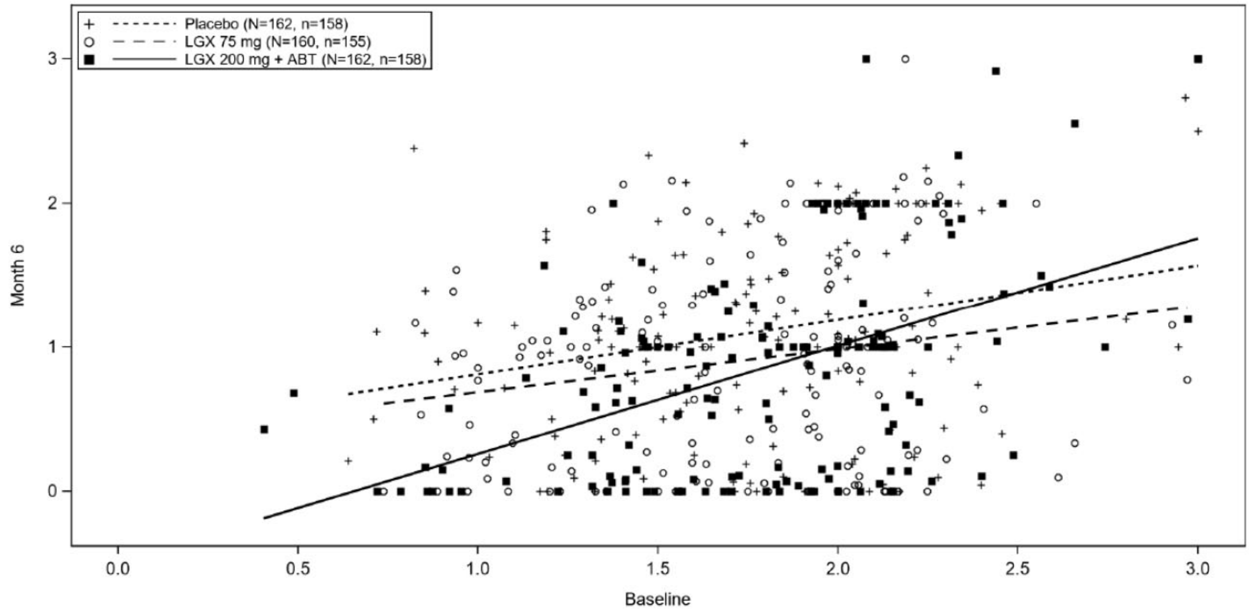

LGX: LINZAGOLIX; ABT: Add-Back Therapy; VRS: Verbal Rating Scale.

Supplementary Figure S1. Month 6 scores: mean of daily assessments in the last 28 days prior to Month 6 or discontinuation. (**A**) Dysmenorrhea and (**B**) Non-menstrual pelvic pain. Due to the large sample size and discreteness of outcomes, a small random variation was added to avoid over-plotting and/or random sub-sampling.
