# Supplementary material for: Linzagolix therapy versus a placebo in patients with endometriosis-associated pain: a prospective, randomized, double-blind, Phase 3 study (EDELWEISS 3)
Source: Hum Reprod. 2024 Apr 22;39(6):1208–21. doi: 10.1093/humrep/deae076 (PMC11144970; doi:10.1093/humrep/deae076)
Supplement: deae076_Supplementary_Table_S1 [file deae076_supplementary_table_s1.pdf]

Human Reproduction, 2024, 1–2
https://doi.org/10.1093/humrep/deae076
Supplementary Data

Supplementary Table S1. Inclusion and exclusion criteria.

Inclusion criteria

1. The subject had to provide written informed consent prior to any study-related procedures.
2. The subject had to be a female aged 18–49 years inclusive.
3. The subject had to have her most recent surgical and—if available—histological diagnosis of pelvic endometriosis (laparoscopy, laparotomy, vaginal fornix, or other biopsy) up to 10 years before screening.
4. The subject had to agree to the washout intervals for prohibited therapies (if applicable).
5. The subject had to agree to switch from her usual analgesic rescue medication to only those permitted by the protocol during the screening, treatment, and FU period.
6. The subject had moderate-to-severe EAP during the screening period defined as:
   a) At the screening visit, a score of at least 2 for DYS and at least 2 for NMPP for the previous month assessed with the modified Biberoglu & Behrman (mB&B) scale.
   b) Over two full menstrual cycles (i.e. from Day 1 of the first menstruation going over 2 spontaneous menstrual cycles up to the day before the next menstruation, i.e. the third menstruation) finishing just before the baseline visit:
      i) Mean overall pelvic pain (OPP) scores of at least 4 on the 0–10 Numeric Rating Scale (NRS) over the 5 days with the highest score for each cycle separately, i.e. required for both cycles.
      ii) At least 2 days with 'moderate' or 'severe' pain on the 0–3 Verbal Rating Scale (VRS) for pelvic pain over the days with uterine bleeding for each cycle separately, i.e. required for both cycles.
      iii) At least 2 days with 'moderate' or 'severe' pain on the 0–3 VRS for pelvic pain over the days without uterine bleeding for each cycle separately, i.e. required for both cycles.

7. The subject had to be compliant with eDiary completion, i.e. has completed at least 75% of days during the Screening period.
8. The subject had regular menstrual cycles and the total length of the two screening menstrual cycles should have been between 42 and 76 days inclusive.
9. The subject had a BMI ≥18 kg/m^2 at the screening visit.
10. If of childbearing potential, the subject agreed to use one of the following birth control methods during the screening period, the entire treatment period of the study, and until 3 months after the end of treatment:
    a) Sexual abstinence, if this is the subject's habitual practice and/or the subject is routinely abstinent from heterosexual intercourse,
    b) Partner with a vasectomy with confirmed azoospermia,
    c) Double non-hormonal barrier contraception such as condom or diaphragm each combined with spermicide.

11. If of non-childbearing potential, the subject had to have had tubal ligation sterilization at least 2 months before the Screening visit.
12. Subjects ≥40 years of age at the screening visit had to have a normal mammogram within 1 year before randomization.
13. The subject had to be able to communicate well with the Investigator and research staff and to comply with the requirements of the study protocol.

Exclusion criteria

1. The subject was pregnant or breastfeeding or was planning a pregnancy within the duration of the treatment period of the study.
2. The subject was <6 months postpartum or 3 months post-abortion/miscarriage at the time of entry into the screening period.
3. The subject had a surgical history of:
   a) Hysterectomy,
   b) Bilateral oophorectomy,
   c) Vagotomy, bowel resection, or any surgical procedure (including gastric surgery) that might interfere with gastrointestinal motility, pH, or absorption,
   d) Any major abdominal surgery (including laparotomy for endometriosis) within 6 months or any interventional surgery for endometriosis performed within a period of 2 months before Screening, or the subject was scheduled for a surgical abdominal procedure during the course of the study.

4. The subject had a tubal sterilization which was performed with ESSURE™.
5. The subject had endometrial ablation resulting in amenorrhea.
6. The subject had at least one ovarian endometrioma with a diameter of 7 cm or greater.
7. The subject was likely to require treatment during the study OR received treatment within a specified period prior to Screening with any of the medications listed below:

a.	GnRH antagonists	3 months
b.	GnRH agonist injections/3-month depot injections	3/6 months
c.	Danazol	3 months
d.	Oral contraceptives and other sex hormones	1 month
e.	Depot contraceptives	10 months
f.	Selective progesterone receptor modulators, selective estrogen receptor modulators, and aromatase inhibitors	3 months
g.	Long-acting narcotics (i.e. requiring less than once daily dosing)	1 day
h.	Systemic glucocorticoid treatments for acute diseases (not depot)	1 month
i.	Medical (prescribed) marijuana	1 week
j.	*In situ* copper intrauterine device (IUD)	1 day
k.	*In situ* IUD with progestogen	1 month

8. The subject was likely to use cannabinoids during the study washout, screening, or treatment period.
9. The subject had required more than 2 weeks of continuous use of narcotic analgesics for treatment of EAP within 6 months prior to screening.

(continued)

Supplementary Table S1. Continued

Inclusion criteria
10. The subject received strong cytochrome P450 (CYP) 3A4 inducers or inhibitors that (might potentially) interact with ABT within 1 month prior to randomization.
11. The subject had a contraindication to ABT including:<br>a) Active deep vein thrombosis, pulmonary embolism, or history of these conditions;<br>b) Active or recent (e.g. within the past year) arterial thromboembolic disease (e.g. stroke, myocardial infarction);<br>c) Known, suspected, or history of breast cancer;<br>d) Known or suspected estrogen-dependent neoplasia;<br>e) Known protein C, protein S, antithrombin deficiency, or other known thrombophilia disorders, including Factor V Leiden;<br>f) Migraine with aura;<br>g) History of porphyria;<br>h) Known hypersensitivity to the ingredients.
12. The subject had a history of current systemic glucocorticoid therapy for the treatment of chronic diseases (e.g. systemic lupus erythematosus, rheumatic arthritis). Inhaled glucocorticoids, e.g. for asthma, were not considered systemic glucocorticoids.
13. The subject did not respond to prior treatment with GnRH agonists or GnRH antagonists for endometriosis.
14. The subject had alanine aminotransferase (ALT), aspartate aminotransferase (AST), total bilirubin (TBL) levels or gamma-glutamyl transpeptidase (GGT) level 2 times the upper limit of normal (ULN), and indicative of potential liver damage at Screening or Day 1 (subjects with abnormalities at Day 1 were to be withdrawn from the study on receipt of the results).
15. The subject had clinically significant abnormal electrocardiogram (ECG), or ECG with QTc using Fridericia's correction formula (QTcF) >450 msec at Screening or Day 1 (prior to dosing).
16. The subject had a known positive human immunodeficiency virus (HIV) or viral hepatitis serology.
17. The subject had abnormal uterine bleeding of undiagnosed cause.
18. The subject had clinically significant findings from a Papanikolaou (PAP) smear test performed within the past 12 months or at the Screening visit which would require surgical intervention (e.g. loop electrosurgical excision procedure or cervical conization).
19. The subject had chronic pelvic pain that, in the opinion of the Investigator, was not caused by endometriosis and required chronic analgesic or other chronic therapy which would have interfered with the assessment of EAP (e.g. interstitial cystitis, presumptive adenomyosis, fibroids, non-endometriosis-related pelvic adhesive disease, post-tubal ligation, or irritable bowel syndrome).
20. The subject had any other clinically significant gynecological condition identified during screening transvaginal ultrasound (TVUS) or endometrial biopsy which might have interfered with the study efficacy and safety objectives (e.g. endometritis, endometrial hyperplasia). However, uterine fibroids (as long as uterus size ≤12 weeks, i.e. equivalent gestational weeks) and adenomyosis were allowed provided they did not interfere with the assessment of EAP (see previous criterion).
21. The subject had any known condition, including findings in the medical history or in the screening assessments, which in the opinion of the Investigator constituted a risk or a contraindication to the participation of the subject in the trial or that could have interfered with the trial objectives, conduct, or evaluation.
22. The subject had a history of, or known, osteoporosis, hyperparathyroidism, or other metabolic bone disease.<br>a) Screening DXA results of the lumbar spine (L1–L4), femoral neck, or total hip BMD showed a Z-score ≤–1.5;<br>b) Any condition that would have interfered with obtaining adequate DXA measurements (e.g. weight [>300 pounds or 136 kg], history of spinal surgery, spinal hardware, severe scoliosis);<br>c) Intercurrent bone disease;<br>d) History of hip fracture;<br>e) History of pathologic or compression fractures; and<br>f) History of bilateral hip replacement.
23. The subject had a mental condition rendering her unable to understand the nature, scope and possible consequences of the study, or evidence of an uncooperative attitude.
24. The subject had a current problem with alcohol or drug abuse (including painkiller abuse).
25. The subject had been administered with any experimental drug in the 12 weeks before Screening.
26. The subject had a calcium level above the ULN range at screening, which was confirmed on repeat fasting testing at screening.
27. The subject had a history of, or active malignancy (with or without systemic chemotherapy) (except treated basal carcinoma of the skin which was not an exclusion criterion).
28. The subject had a history of attempted suicide and/or a history of, or known major psychiatric disorders that were not well controlled.
